# Dynamic Ultrasound Uniquely Reveals Anterior Capsular Tear Communicating With Vastus Intermedius Fibers

**DOI:** 10.7759/cureus.8880

**Published:** 2020-06-28

**Authors:** Shiv J Patel, Jasvinder A Singh, Max H Epstein, Prathap Jayaram

**Affiliations:** 1 H. Ben Taub Department of Physical Medicine and Rehabilitation, Baylor College of Medicine, Houston, USA; 2 Joseph Barnhart Department of Orthopedic Surgery, Baylor College of Medicine, Houston, USA

**Keywords:** capsular tear, knee pain, vastus intermedius, patellar tendon, quadriceps pain, tendinopathy, patellar tendinopathy, dynamic ultrasound, knee ultrasound

## Abstract

We present a case of an anterior capsular tear of the right knee in a previously healthy, active individual. The patient was a 31-year-old male seen one month after the onset of throbbing anterior right knee pain, which progressed to sharp suprapatellar pain over the next three months. Both dynamic ultrasound (US) and MRI were obtained. Dynamic US revealed a tear of the anterior capsule of the right knee complicated by a suprapatellar effusion communicating into the vastus intermedius fibers of the quadriceps tendon. However, these findings were not evident on MRI. In addition to discussing this unique pathology, we highlight the utility of both standard and dynamic US in establishing diagnoses for capsular pathologies.

## Introduction

Currently, there are studies in the literature that report capsular tears of the knee concomitant with the rupture of stabilizing tendons, but there are no studies, to our knowledge, that describe anterior capsular tears in the absence of tendon rupture. Furthermore, there were no studies in our literature review investigating the effectiveness and capability of dynamic ultrasound (US) in assessing capsular tears of the knee in relation to MRI and the standard US. Dynamic US involves manipulating the target extremity while maintaining visualization of affected structures to observe behavior of the target under varying conditions such as flexion or extension. Prior studies evaluating pathologies such as rotator cuff tears have not suggested a significant difference between MRI and the standard US [1,2]. This case demonstrates, similarly, that the dynamic US may be a useful tool for rapidly and cost-effectively evaluating connective tissue injuries of the knee.

## Case presentation

**Figure 1:**
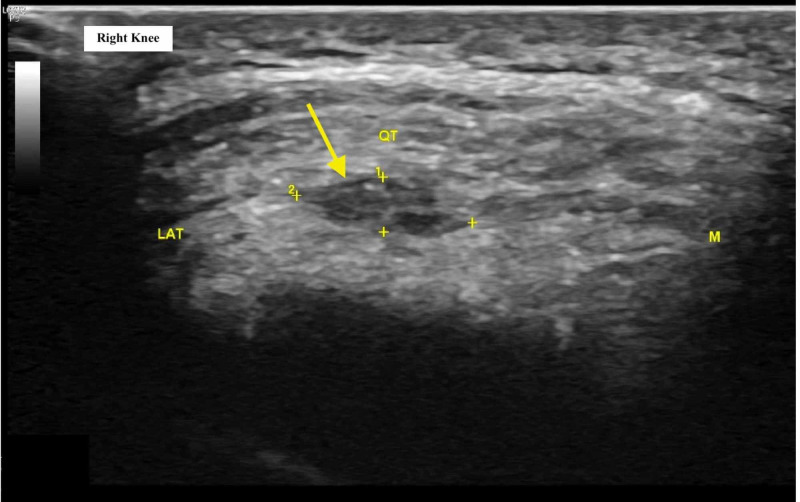
Short-axis ultrasound image of the right knee at initial visit demonstrating evidence of quadriceps tendinopathy, micro tears, calcific enthesopathy, and intrasubstance hypoechogenicity (yellow arrow) LAT: lateral anatomic orientation; M: medial anatomic orientation; QT: quadriceps tendon

A 31-year-old active male with no prior medical history presented to the clinic with a one-month history of chronic, intermittent, throbbing, anterior-inferior right knee pain with severity rated 2/10 on average. The pain was aggravated by squatting and lunging and relieved with rest and icing. The patient reported no family history of musculoskeletal disorders and no history of substance abuse. On physical examination, the patient had a general athletic appearance and muscle tone was normal and symmetric bilaterally throughout both lower extremities. The right knee was observed to have a decreased range of motion to knee extension. Standard US of the right quadriceps tendon revealed hypoechogenicity and evidence of a partial tendon intrasubstance tear (Figure 1). Conservative management (rest, ice, compression, elevation) and physical therapy were recommended.

**Figure 2:**
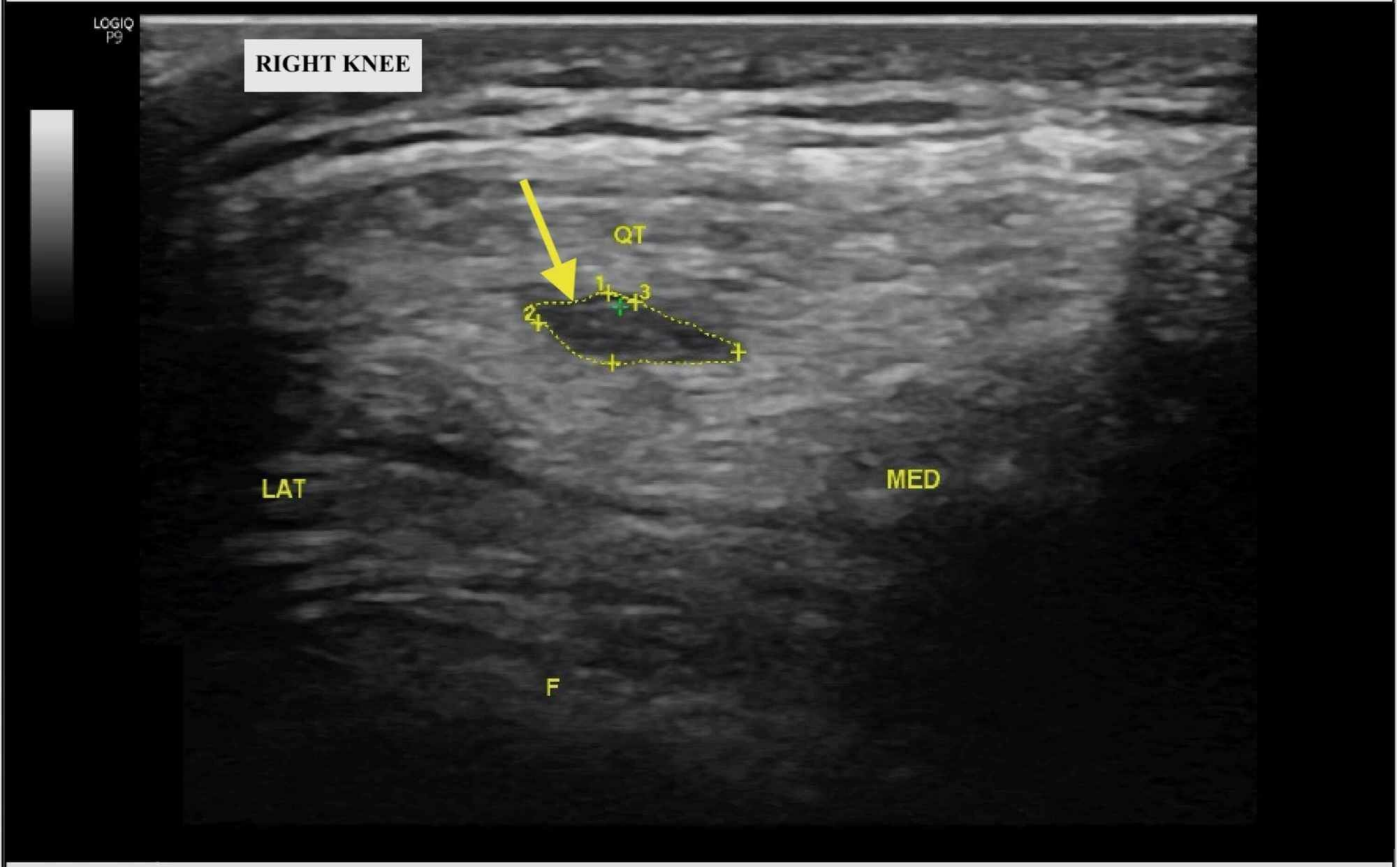
Short-axis ultrasound image of the right knee at three-month follow-up demonstrating hypoechogenicity within the right quadriceps tendon (yellow arrow) LAT: lateral anatomic orientation; F: femur; MED: medial anatomic orientation; QT: quadriceps tendon

**Figure 3:**
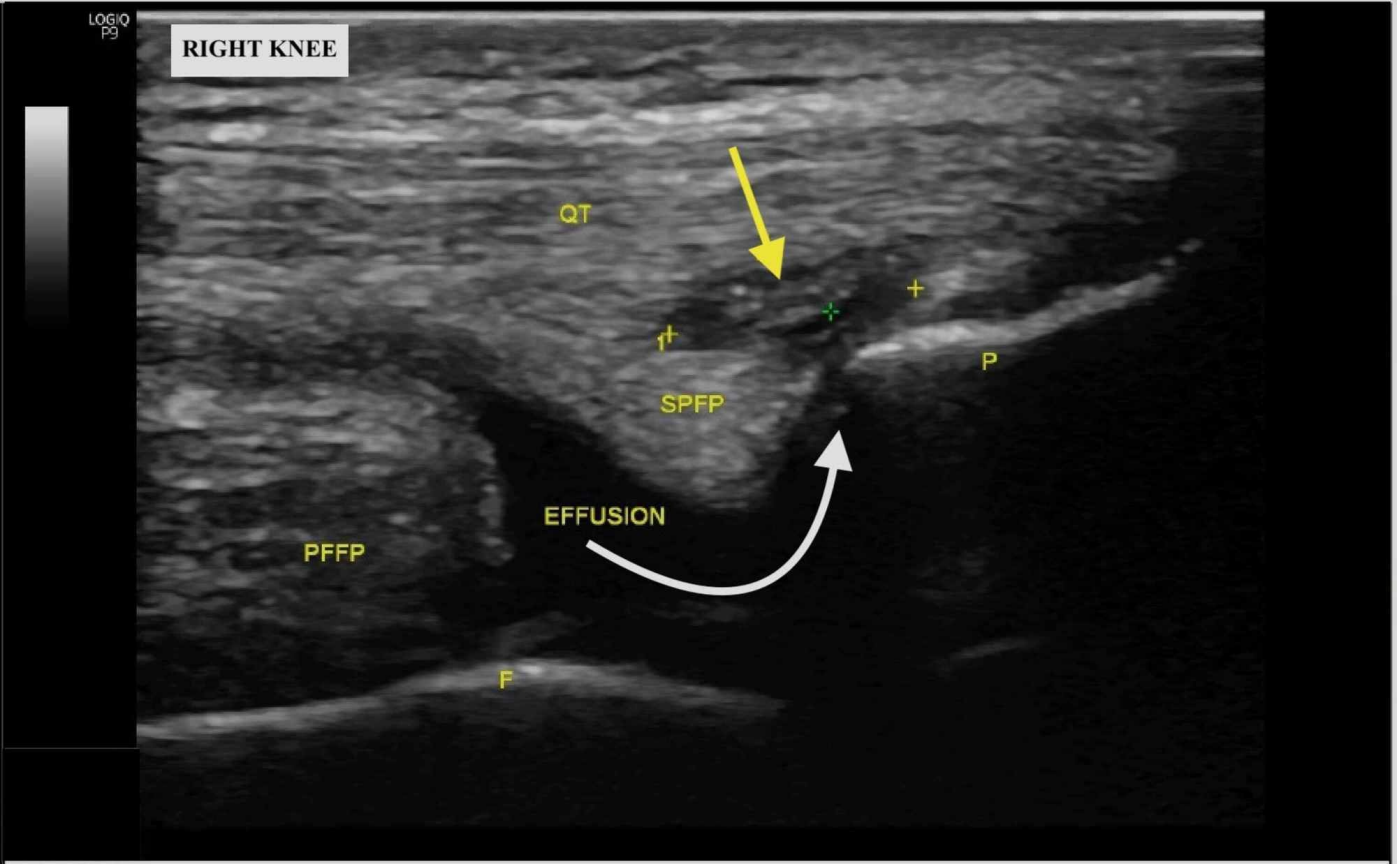
Long-axis ultrasound image of the right knee at three-month follow-up; white arrow demonstrates the path of a suprapatellar effusion communicating into the vastus intermedius fibers of the right quadriceps tendon (yellow arrow) QT: quadriceps tendon; PFFP: prefemoral fat pad; SPFP: suprapatellar fat pad; F: femur; P: patella

At three-month follow-up, the patient reported significant improvement of the right anterior-inferior knee pain but reported new right suprapatellar pain. This pain was sharp and intermittent with no associated swelling and exacerbated with running. Repeat standard US of the right knee in the anterior approach with a longitudinal view revealed a new suprapatellar effusion, hypoechoic intrasubstance tendinopathy, and calcific enthesopathy of the quadriceps tendon (Figure 2). MRI corroborated these findings but demonstrated nothing additionally. However, dynamic US revealed an anterior capsular tear with a suprapatellar effusion communicating into the vastus intermedius fibers of the quadriceps tendon when the knee was positioned in extension (Figure 3). A plain film radiograph demonstrated patella alta, confirmed using the Blackburne-Peel Index [3]. We recommended continued conservative management, and at the final follow-up one month later, the patient expressed significant resolution of symptoms [4].

## Discussion

There exists a noticeable dearth of literature comparing the diagnostic accuracy of ultrasound to MRI in pathologies involving the knee joint capsule. However, the value of dynamic US in evaluating other musculoskeletal pathologies has been documented in the literature. Multiple studies have found there to be no significant difference between MRI and US in accurately diagnosing partial and full-thickness rotator cuff tears [1,2]. In addition, another study demonstrated that dynamic US had the adequate capability to diagnose adhesive capsulitis of the shoulder with a 92% accuracy [5]. The capsular tear described in this case may have been caused by this patient’s involvement in high impact exercises such as high jumps and CrossFit training. Furthermore, this patient’s patella alta may have catalyzed this injury because the longer patellar tendon results in an upward displacement of the patella and greater baseline patellar instability [4,6]. Critically, the capsular tear was only visible on the dynamic US as MRI failed to show the communication. The salient takeaway, in this case, is the profound utility of dynamic US in establishing an initial and accurate diagnosis of a capsular tear of the knee joint. We were able to pursue a directed, conservative approach based on standard US findings in the first clinic visit, which was solidified in the follow-up visit via the use of dynamic US. An MRI was obtained, but no additional information was revealed and in fact, did not reveal the capsular tear. For these reasons, we suggest an investigation of the benefit of dynamic US compared to MRI in various joints, especially in the knee, in future studies.

## Conclusions

The initial management of new knee pain has traditionally called for the use of a plain film X-ray or MRI. More recently, dynamic US has added a new dimension to a standard imaging modality as it is readily performed in the clinic setting at the time of presentation and can provide position-dependent visualization of pathology. We demonstrate with this case that the dynamic US is a valuable point of care diagnostic tool in the evaluation of anterior capsular tears and extra-articular effusions. Furthermore, we show that the dynamic US is expedient, low cost, and sometimes more accurate diagnostic imaging tool than classic modalities such as MRI when evaluating knee-pain.
